# Is Lipid Metabolism of Value in Cancer Research and Treatment? Part I- Lipid Metabolism in Cancer

**DOI:** 10.3390/metabo14060312

**Published:** 2024-05-29

**Authors:** Ala F. Nassar, Xinxin Nie, Tianxiang Zhang, Jacky Yeung, Paul Norris, Jianwei He, Hideki Ogura, Muhammad Usman Babar, Anne Muldoon, Stephania Libreros, Lieping Chen

**Affiliations:** 1 Department of Immunobiology, Yale University, West Haven, CT 06516, USA; 2 Sciex, 500 Old Connecticut Path, Framingham, MA 01701, USA; 3 Department of Microbiology, Hyogo Medical University, Nishinomiya 663-8501, Japan; 4 Department of Pathology, Yale University, New Haven, CT 06520, USA; 5 Vascular Biology and Therapeutic Program, Yale University School of Medicine, New Haven, CT 06520, USA

**Keywords:** lipids, lipid metabolism, cancer, ion-mobility mass spectrometry (MS), immunity, lipidomics, MS imaging

## Abstract

For either healthy or diseased organisms, lipids are key components for cellular membranes; they play important roles in numerous cellular processes including cell growth, proliferation, differentiation, energy storage and signaling. Exercise and disease development are examples of cellular environment alterations which produce changes in these networks. There are indications that alterations in lipid metabolism contribute to the development and progression of a variety of cancers. Measuring such alterations and understanding the pathways involved is critical to fully understand cellular metabolism. The demands for this information have led to the emergence of lipidomics, which enables the large-scale study of lipids using mass spectrometry (MS) techniques. Mass spectrometry has been widely used in lipidomics and allows us to analyze detailed lipid profiles of cancers. In this article, we discuss emerging strategies for lipidomics by mass spectrometry; targeted, as opposed to global, lipid analysis provides an exciting new alternative method. Additionally, we provide an introduction to lipidomics, lipid categories and their major biological functions, along with lipidomics studies by mass spectrometry in cancer samples. Further, we summarize the importance of lipid metabolism in oncology and tumor microenvironment, some of the challenges for lipodomics, and the potential for targeted approaches for screening pharmaceutical candidates to improve the therapeutic efficacy of treatment in cancer patients.

## 1. Introduction

Lipids, which are fatty mixtures of fats, waxes, sterols, fat-soluble vitamins (vitamins A, D, E and K, for example), monoglycerides, diglycerides, triglycerides and phospholipids, achieve a range of functions in our body. Those lipids, which are part of the cell membranes, serve to help control what goes in and out of our cells, as well as helping with moving and storing energy, absorbing vitamins and making hormones. Having an excessive amount of some lipids is harmful; a lipid panel assay can tell us if a person has normal levels. Hundreds of thousands of individual lipid molecular species are present in cells. Phospholipids represent the bulk of lipids in mammalian cells, accounting for about 60 mol% of the total, while glycolipids and sphingolipids combine to account for about 10 mol%. The distribution of non-polar lipids, among which are triacylglyceride (TAG) and cholesterol, varies among cell types and compartments, ranging from 0.1 to 40 mol%. Natural metabolites form within the cell, generally comprising less than 5 mol% of total lipids. This group includes non-esterified fatty acids (NEFAs, or free fatty acids), lysolipids, diacylglycerol, ceramides, acyl carnitines, and acyl CoA. Excessive levels can produce harmful pathophysiologic sequelae under a variety of pathologic conditions [1,2,3,4].

**Figure 1:**
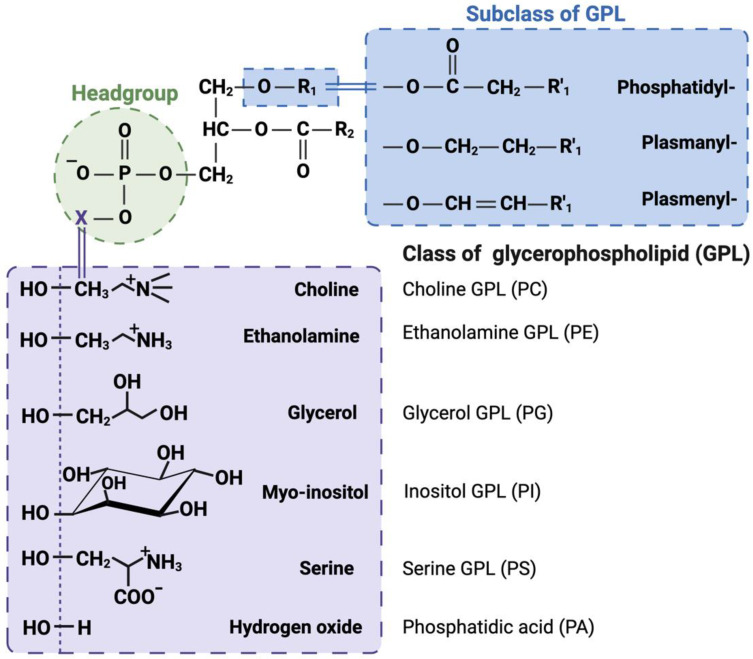
Summary of classes, subclasses and molecular species in glycerophospholipids.

The lipidome represents the total lipid content in a cell; the variety of these lipids is predicted to be in the range of tens of thousands to hundreds of thousands, with a wide range of concentrations. There are several classes and subclasses of lipids, characterized by the head group and the type of linkage between the head group and aliphatic chains (Figure 1) [5].

## 2. Lipid Categories and Their Major Biological Functions [5,6,7,8,9,10,11]

Based on their chemical structure, lipids have been classified into eight categories, as shown in Table 1 [11]:

- Fatty acyls: The molecules in fatty acyl groups are synthesized by chain elongation of acetyl coenzyme A (CoA) primers with malonyl–CoA groups. They may contain cyclic functional groups and/or be substituted by heteroatoms. The structure of fatty acyls is the simplest, as they are characterized by a repeating series of methylene groups. Given their simplicity, fatty acyls function as the basic components of other, more complex lipids.
- Prenol lipids: Five-carbon isoprene units represent the basic building blocks for prenol lipids. They may contain linear, cyclic or branched isoprene chains, any of which may consist of fatty acids, characteristic side-chains, functional groups, sugars, and double bonds. Given the range of permutations, these basic structures may form thousands of unique lipids. Prenol lipids are based on one or multiple five-carbon isoprene units, the building blocks of prenol lipids. The isoprene chain can be linear, cyclic or branched and features fatty acids, characteristic side-chains, functional groups, sugars and double bonds. The flexibility of the prenol lipids’ structure gives rise to thousands of different lipid species.
- Glycerolipids: The glycerolipid groups include monoacylglycerol, diacylglycerol, triacylglycerol (TAG) and glycolipids, and they are characterized by the presence of a glycerol backbone with fatty acyl chains connected to the hydroxyl groups of glycerol. Glycerolipids can be hydrolyzed into glycerol, non-esterified fatty acid (NEFA) and/or alkyl variants; they contribute to energy storage, energy metabolism and signal transduction.
- Glycerophospholipids: Glycerophospholipids (GPLs) contain at least one glycerol hydroxyl group esterified with one phosphate or phosphonate group. This category includes phosphatidylcholine (PC), phosphatidyl ethanolamine (PE), phosphatidylinositol (PI), phosphatidylserine (PS), phosphatidylglycerol (PG), phosphatidic acid (PA), cardiolipin, bis(monoacylglycerol) phosphate and lysophospholipids, etc. GPLs represent the bulk of cellular and plasma membranes and are one of their key components. GPLs contribute to second messenger generation and are involved in cellular metabolism and signal transduction.
- Sphingolipids: Sphingolipid groups act to control cellular signaling; they are components of plasma membranes. They are characterized by their core structure, specifically long-chain sphingoids such as ceramide (Cer), sphingomyelin, cerebroside, sulfatide and gangliosides.
- Saccharolipids: The saccharolipids are a group of lipids which contain a sugar backbone directly connected with fatty acids (as opposed to the glycerol backbone in glycerolipids and glycerophospholipids). They exist of glycans or phosphorylated derivative forms.
- Sterol lipids: Sterol lipids are compounds containing four fused rings. Among the subcategories of sterol lipids are cholesterol and its derivatives, steroids, bile acids and their derivatives, etc. Cholesterol, along with its derivatives, is a vital component of cellular membranes. Steroids function as hormones and signaling molecules and are important to a variety of additional biological and metabolic processes.
- Polyketides: The polyketides are a group of metabolites with characteristics similar to lipids. They come from plant and microbial sources. This category of lipids functions as cellular membrane components, energy storage depots and signaling molecules (Table 1).

It is well known that lipid metabolites alter tumors; indeed, this is considered a hallmark of cancer. There is a growing body of evidence that tumor growth is promoted through lipid metabolism deregulation; furthermore, it aids in cell migration, invasion and angiogenesis. Table 2 presents a list of the major functions of individual lipid classes including membrane components, energy storage and metabolism, signaling and other functions such as having antioxidant activity, acting in lipid transport and mitochondrial respiration, and as a cofactor and substrate of PE synthesis. For example, cholesterol defines and regulates the function and extent of lipid rafts on membranes. Some glycerolipids, such as NEFA, acylcarnitine, acyl CoA, diacylglycerol and TAG, are involved in energy metabolism and act as energy storage depots. Table 2 provides a summary of lipids contributing to signaling regulation. 

## 3. Lipidomics Studies in Cancer Samples (MS Tech) 

### 3.1. Sample Preparation and Lipid Extraction

**Figure 2:**
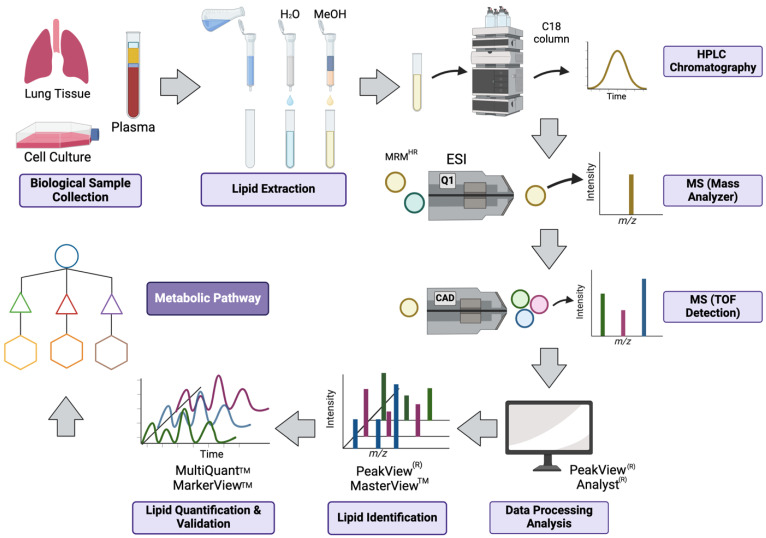
Experimental workflow of lipid mediators analysis using MRM HR.

The wide and varied range of lipid classes precludes the use of a standard, one-size-fits-all extraction technique. A generalized experimental workflow for lipid analysis is shown in Figure 2 [12]. As always, prior to analysis, the samples must be prepared, stored and processed properly. Fast, reproducible preparation methods which are able to handle various lipids and their different polarities while maintaining compatibility with the instrumental technique are ideal. Three major techniques are liquid–liquid extraction, organic solvent precipitation and solid-phase extraction, with organic solvent precipitation methods being the most common; each offers high rates of lipid recovery for lipodomics researchers. An early standard, the Bligh and Dyer procedure, involves chloroform–methanol based protocols which produce phase partitioning into the organic layer. More recent protocols have improved this method, producing better targeted lipid isolation and increased high-throughput data collection. While these methods work well for physiologically relevant lipids, one remaining problem area involves analysis of low-abundance and labile lipid metabolites [12,13,14,15,16,17].

**The common extraction methods used in lipidomics**

- Modified Bligh and Dyer. Chloroform/methanol/H_2_O (1:1:0.9, *v:v:v*) for extraction of a small amount of biological sample (e.g., <50 mg of tissue). It is well-established and broadly practiced. The disadvantages include the use of hazardous chloroform and the collection of chloroform extract from the bottom layer, which may cause the carry-over of water-soluble impurities and difficulty in automation.
- Modified Folch. Chloroform/methanol (2:1, *v:v*), 0.9% NaCl (0.2 volume) is used to extract biological tissue (e.g., ~0.1 g). Water or 0.9% NaCl (0.2 volume) is then added to wash the solvent extract. It has similar advantages and disadvantages to the modified Bligh and Dyer method.
- Methyl tert-butyl ether (MTBE) method. The ratios are MTBE/methanol/water (5:1.5:1.45, *v:v:v*). This method resolves some of the difficulties in chloroform-involved methods because MTBE is present in the top layer after phase separation and therefore is more feasible for high throughput and automation. A disadvantage is that the MTBE phase contains a significant amount of aqueous components that may produce carry-over of water-soluble contaminants.
- Butanol/methanol method. Start with a volume of butanol/methanol (BUME, 3:1, *v*/*v*) added to a small volume of aqueous phase. Next, an equal volume of heptane/ethyl acetate (3:1, *v*/*v*) is added, and then 1% acetic acid is added (equal volume to BUME) to induce phase separation. This method may compensate for the issue with the previous method, with less water-soluble contaminants being carried over in the organic phase. Its drawback is the difficulty in evaporation of the butanol component in the organic phase.

### 3.2. Lipid Separation by HPLC

For separating lipids before mass analysis, high-performance liquid chromatography (HPLC or LC) is a common choice. There are several means of separation, including Hydrophilic Interaction Liquid Chromatography, supercritical fluid chromatography or two-dimensional liquid chromatography. Each of these has shown to be suited for particular uses: NP-HPLC is most effective to separate glycerophospholipids using headgroup polarity, while RP-HPLC uses chain length, degree of unsaturation and substitution to sort out fatty acids like Eicosanoids. The combination of RP and NP or Hydrophilic Interaction Liquid Chromatography (HILC) columns provides wider lipidome coverage for untargeted studies. For general lipodomics studies, nano-flow liquid chromatography (nLC) works to improve lipidome coverage and, in general, the sensitivity of measurements. When the eluate is integrated with the ionization source of a mass spectrometer, online or offline chromatographic (HPLC/UHPLC) separation of lipids is a good choice [18,19,20,21,22,23,24,25,26,27,28,29,30,31,32,33,34,35,36,37,38,39,40,41,42,43,44,45].

- Reversed-phase LC. This type of LC works by separating lipids based on their hydrophobicity. Although retention times vary between individual lipids, this remains the most commonly used method for working with complex lipids. Lipidomics research generally employs short columns (range of 50–150 mm; typically, 100); particle sizes either less than 2 µm or fused-core between 2.6 and 2.8 µm; and sorbents modified with C18 or C8
- Normal-phase LC. This type of LC is a good complement to reversed-phase LC. It uses highly non-polar solvents with low ionization capacity to separate lipids based on their polarities, which makes it an excellent separation mechanism for phospholipids, especially PA. With its longer retention times, generally between 30 and 60 min, it improves separation of large particle sizes on long columns. Lipid classes may be separated using this technique.
- Hydrophilic interaction liquid chromatography. This technique is more reproducible and robust, with the added benefit of greater compatibility with MS. However, the physical properties of either reversed-phase or normal-phase columns are somewhat compromised.
- Supercritical fluid chromatography. Using columns packed with sub-2 µm particles, this method is a newly emerging technique that can be used for fast lipid profiling (<20 min).
- Two-dimensional liquid chromatography. In this technique, the injected sample is separated by passing through two different separation stages. This is accomplished through sequentially connecting two different chromatographic columns, with the effluent from the first system being transferred onto the second column. It can optimize the separation conditions of complex lipidomes. One drawback is that it requires more labor and time.

### 3.3. Summary of Modern MS-Based Lipidomics Approaches (Table 3)

- LC-MS/MS

Resolves lipid classes and/or molecular species before MS analysis.

Uses pre-separation of lipids.

Powerful for targeted analysis of very low abundance lipid classes, but slow for comprehensive analysis of cellular lipidomes.

- Shotgun lipidomics

Uses lipid chemistry and physics for analysis under constant concentrations.

Fairly high throughput, but dynamic range might be affected with coexistence of other major lipids.

- MALDI-MS

Provides rapid screening and can identify lipid classes with high sensitivity.

Comprehensive analysis is limited.

- MS imaging

Useful for determining spatial distribution of lipids.

Largely qualitative comparison.

- Ion-mobility MS

A post-ionization separation technique based on the mass, charge, size and shape of lipids.

Enables class separation analysis of isomeric and isobaric species.

**Mass Spectrometry Lipid Analysis.**

There are three major categories of MS techniques, each with a different purpose:

1. Global Lipidomic Analysis: This type of approach, referred to as a “shotgun” technique, offers the capability to identify and quantify large numbers of cellular lipids on a high-throughput basis. Several of these techniques are widely used for analyses of wide-ranging pathways and networks involved with metabolism, trafficking and homeostasis of lipids. Recent developments in mapping techniques provide support for research into spatial and temporal relationships among lipids.
2. These methods use LC-MS and LC-MS/MS to identify a Targeted Lipidomic Analysis: much smaller set of lipids of interest.
3. Novel Lipid Discovery: Techniques using LC coupled with MS work with different enrichment technologies with the aim of finding novel classes and molecular species of lipids. This type of analysis involving lipid mediators in biological samples is complicated by their low concentrations in biological samples, their transient nature and limited half-lives.

Targeted versus untargeted approaches. Untargeted analyses are preferred for producing the widest possible scan of lipid ions, useful for examining the lipid composition for discovery efforts. However, these studies are hindered by the limitations of TOF and Orbitrap analyzers, including lower sensitivity and quantitative abilities. Due to this issue, targeted approaches, which involve a predefined list of lipid species of interest, are often used instead. This difficulty is overcome through the use of quadrupole instruments, which apply a specific sequence of voltages, ranging from a few to several thousands, on the mass filters correlated to the lipids of interest. Such ‘multiple reaction monitoring’ allows for more sensitive and accurate quantification of each individual species.

**Table 3:** MS-based lipidomics approaches.

Parameter	Type of MS				
	Shotgun MS	LC-MS/MS	MALDI-MS	MS Imaging	Ion-Mobility MS
Reproducibility	High	High	Medium	Medium	High
Sensitivity	Low	High	Medium	Medium	High
Resolution	Low	High	High	High	High
Quantitativity	Yes	Yes, with IS	Yes	Yes	Yes, with IS
Sample preparation	Minimal	Extensive sample preparation steps	Minimal	Minimal	Extensive sample preparation steps
Structural information	Yes	Yes	No	No	Yes
High throughput	Yes, but dynamic range might be affected with coexistence of other major lipids	Yes	No	No	Yes
Information/Key point	- Uses lipid chemistry and physics for analysis under constant concentrations - Infuse sample directly into the MS - Less time-consuming and low cost - High reproducibility - Low sensitivity incapable of distinguishing isomers - Application-typical building blocks include glycerol, sphingoid bases, polar head groups and fatty acyl substituents (or other aliphatic chains).	- Uses pre-separation of lipids - Resolves lipid classes and/or molecular species before MS analysis - Powerful for targeted analysis of very low abundance lipid class, but fairly slow for comprehensive analysis of cellular lipidomes - The most commonly used method in lipidomics; able to analyze a wide variety of lipids - High separation efficiency and sensitivity - Organic solvent Consumption Metabolite target analysis - Metabolite profiling Lipidomics	- Provides rapid screening and can identify lipid classes with high sensitivity - Comprehensive analysis is limited	- Useful for determining spatial distribution of lipids - Largely qualitative comparison - Ionize sample by coating with matrix and irradiating laser - High resolution; the most established technique - Requires matrix pretreatment	- A post-ionization separation technique based on the masses, charges, sizes and shapes of lipids - Might enable the analysis of isomeric and isobaric species - Comprehensive analysis under development

LC–MS, liquid chromatography–mass spectrometry; IS, internal standard. MALDI, matrix-assisted laser desorption/ionization; MS, mass spectrometry, HPLC, high-performance liquid chromatography; MSI, mass spectrometry imaging.

### 3.4. Ionization Technique

- ESI MS. This technique works with the gaseous ions generated from polar, thermally labile and mainly non-volatile molecules and is useful for a variety of lipids. As a soft-ionization method, its effects on the sample chemistry are minimal prior to analysis. There are a number of ESI-MS techniques which enable analysis of a variety of classes, subclasses and individual lipid species within biological extracts. One technique, referred to as “shotgun lipodomics”, has been developed by Han and his team. For this method, a crude lipid extract is infused directly into an ESI source which is tuned for separation of lipids using their intrinsic electrical properties.
- APCI MS. While it is similar to ESI, this method relies on the generation of ions as the heated analyte solvent interacts with a corona discharge needle set for a high electrical potential. As primary ions form in the immediate vicinity of the needle, their interaction with the solvent forms secondary ions, which, in turn, ionize the sample. This is a very good technique for the analysis of nonpolar lipids such as triacylglycerols, sterols and fatty acid esters.
- DESI MS. DESI mass spectrometry is an ambient ionization technique which combines desorption ionization with ESI. This is accomplished by directing an electrically charged mist to the sample surface that is a few millimeters away, making it an excellent tool for mapping lipid distribution contained in biological samples. A major advantage is that, because no matrix is required for tissue preparation, DESI MS allows for multiple consecutive measurements on the same tissue specimen.
- MALDI MS. This technique, typically employed in the analysis of large proteins, has proven beneficial in lipid studies. A mixture containing a matrix such as 2,5-dihydroxybenzoic acid and the lipid sample is placed on the sample holder in a small spot. When a laser is fired at the spot, the matrix absorbs the energy, subsequently transferring it to the analyte, resulting in ionization of the molecule. MALDI–time-of-flight (MALDI-TOF) MS has shown great promise as an approach for lipidomics studies, particularly for the imaging of lipids from tissue slides.

Spatial lipidomics. With its high sensitivity in the lipid range, DESI is recognized for its strength in the detection and mapping of lipids contained in tissue samples. Additionally, MALDI techniques facilitate direct detection of lipids. When thin tissue slices are coated with such MALDI matrixes, the result is an abundance of lipid-related ions over a sequence of spectra. Detection of phospholipids, sphingolipids and glycerolipids is enhanced in tissues such as heart, kidney and brain. 

The majority of lipidomics measures the relative lipid concentration in an extract of a biological sample such as a human tissue. This analysis generates the average lipid profile of the tissue, not considering regional heterogeneity due to cell-type composition. Spatial lipodomics is required to indicate this heterogeneity. The typical set-up for MALDI analysis involves a thin frozen section of a tissue which is fixed to a slide and covered by a thin layer of matrix. When the slide is then scanned by a laser, lipid ions are released. The separation is measured by the time intervals for the individual ions to travel through a vacuum tube; these intervals are dependent on their mass. This method allows the lipid composition of the tissue to be determined pixel by pixel and visualized in 2D as a colored image of the tissue. The latest high-end instruments enable identification of thousands of lipids at a spatial resolution close to a single cell level (10 μm). In addition to plasma, lipidomic profiling of urine and extracellular vesicles is a newer area of interest which may also prove to be informative as a non-invasive source of cancer biomarkers. Table 4 shows lipidomic studies on biofluids reporting diagnostic lipid signatures [46].

## 4. The Importance of Lipid Metabolism in Oncology

Lipid metabolic reprogramming produces changes in lipid metabolism, which disrupt a variety of cell functions such as cell cycles, proliferation, growth and differentiation; such alterations lead to carcinogenesis. These changes play a significant role in the interaction between the cancer cells and their surrounding microenvironment. There are several types of lipids which, in addition to serving as energy sources and structural components of biological membranes, act as signaling molecules or secondary messengers. Potential lipid biomarkers in the oncology field are shown in Table 5 [47,48,49,50,51,52,53,54,55,56,57,58,59,60,61,62,63,64,65,66,67,68,69,70,71,72,73,74,75,76,77,78,79,80,81,82,83,84,85,86,87,88,89,90,91,92,93,94,95,96].

### 4.1. Fatty Acid (FA)

Lipid uptake is accomplished through membrane-associated transport proteins such as fatty acid transport protein-1 (FATP1), fatty acid translocase (CD36) and liver fatty acid-binding protein (L-FABP). With regard to FA metabolism, their expression is largely regulated by the master transcription factor of lipogenesis, known as sterol regulatory element-binding protein 1 (SREBP-1). Ovarian cancer, gastric cancer, glioblastoma and oral squamous cell carcinoma all involve upregulation of CD-36 expression. While two early drug candidates targeting this process were unsuccessful due to ineffectiveness and severe side effects, newer formulations are currently undergoing preclinical trials. Changes in enzymes involved in FAO (fatty acid oxidation) are common in cancer cells and ultimately affect this major bioenergetic pathway that promotes proliferation, metastasis, stemness and resistance to treatment for a variety of cancers.

Breast cancer, gastric cancer and prostate cancer have been linked with overexpression of carnitine palmitoyl transferase I (CPT1). It was hoped that CPT1 inhibitors such as etomoxir and phexiline would display antitumor activity; severe adverse reactions have derailed further consideration of them. It has been reported that, in patients with nonalcoholic steatohepatitis (NASH), lipotoxicity for hepatocellular carcinoma (HCC) cells is thwarted by CPT2 downregulation-mediated suppression of FAO. Elevated hepatocarcinogenesis, resulting from accumulation of acylcarnitine as an oncometabolite, as well as a reduction in CPT2 in HCC, have been confirmed. There is a negative correlation between CPT2 expression and tumor stage, while low expression of CPT2 is associated with poor prognosis in colon cancer. There are indications that Th17 inflammation in Type 2 diabetes may be activated by FAO. This is achieved through blocking CPT1A or inhibiting FAO, which reduces the production of IL-17 by T helper 17 (Th17) cells.

Because FAO is implicated in the synthesis of many essential fatty acid derivatives, it is considered to play a vital role in cells. For example, fatty acid is the basic material for prostaglandin and leukotriene synthesis. Fatty acid is part of a hot topic of discussion as to whether FAO is required for regulatory T (Treg) cell differentiation. De novo fatty acid synthesis in CD4^+^ T cells depends on monounsaturated fatty acids (MUFAs) for endogenous fatty acid synthesis, with stearoyl-CoA desaturases (SCDs) acting as the catalyst. Strong expression of stearoyl-CoA desaturases (SCDs) is shown in human and murine cells, and mouse models have shown that the inhibition of SCDs leads to Tfh cell apoptosis. Additional mouse experiments indicate that phosopholipid production for cell membranes is facilitated by ACC1-mediated de novo fatty acid synthesis and produces Th17 cell differentiation. Th17 immune response, proliferation and infiltration are suppressed through elimination of ACC1 in murine models of colitis. Further studies reveal correlations between high-fat diets and Th17 cell differentiation; ACC1 similarly affects Th17 cells through added expression of IL17A, IL23R, LTB4R1 and CCR6. There is an abundance of reports in the literature stating that naïve T cell activation and differentiation are dependent on the synergistic relationship between the generation of Acetyl–CoA during glycolysis and de novo fatty acid synthesis.

### 4.2. Cholesterol

Cancer cells require high levels of cholesterol to maintain their rapid proliferation; indeed, increased cholesterol biosynthesis is a hallmark of many cancers. Upregulation of SREBP2 and its target genes is prevalent in prostate cancer, breast cancer and HCC. Activation of the Hedgehog and Notch signaling pathways allows for cholesterol biosynthesis, which, in turn, facilitates cancer stem cell maintenance. Accumulation of cholesteryl esters (CEs) is another characteristic of cancer; for example, Acetyl-CoA acetyltransferase 1 (ACAT1) is correlated with pancreatic cancer and lymphocytic leukemia. Other common components of the tumor microenvironment (TME) include oxysterols; one example is the accumulation of 27-hydroxycholesterol (27HC) in the breast and tumor tissue of estrogen receptor-positive breast cancer patients. One possible anti-tumor therapy involves the inhibition of cholesterol metabolism. Statins, regardless of use before or after diagnosis, are well-known HMGCR inhibitors which reduce mortality in various cancer types and are under clinical investigation as anti-tumor treatments 

HMG-CoA reductase, which is essential for de novo cholesterol synthesis in CD4^+^ T cells, produces mevalonic acid (MVA) from HMG-CoA. The next step along the mevalonate pathway is conversion of MVA to squalene, which is then converted to cholesterol. Acting as an MG-CoA reductase inhibitor by ameliorating hyperlipidemia, statin produces a greater share of Treg cells while suppressing Treg cell formation. With rheumatoid arthritis RA, when Th1 cells are treated with statins, activation of the cholesterol biosynthesis pathway causes them to switch from IFN-γ to IL-10+. This is an indicator that the proinflammatory function of Th1 cells depends on cholesterol biosynthesis. 

### 4.3. Triacylglycerol and Lipid Droplets

Triacylglycerol, or TAG, contains three fatty acids which are esterified to a glycerol molecule; its functions are fatty acid storage and aiding in efficient transport. The rapid proliferation during carcinogenesis increases demand for FA. The production of FA is accomplished through increases in de novo synthesis and uptake from extracellular sources. Autophagy is another source, using lipid droplets to aid in the recycling of intracellular lipids. In addition, lipid droplets are important in defending against reactive oxygen species (ROS) and serve to defend against stress in difficult environments such as hypoxia and low nutrition. There is a correlation between various cancers and excessive concentration of lipid droplets; such accumulations are indicators of a negative prognosis for breast and pancreatic cancers. This shows the value of determining changes in their metabolism. Brain, breast, renal and prostate cancers are all associated with the production of hypoxia-associated lipid droplets. Direct stimulation of Lipin-1 in an HIF-1α-dependent manner results from hypoxia exposure, leading to accumulation of lipid droplets in HCC and cervical cancer. Hypoxia also causes upregulation of FA uptake via FABP3/7 and accumulation of lipid droplets, leading to estrogen receptor (ER) and redox homeostasis during oxygen deprivation. The FA produced during this process is used for mitochondrial energy synthesis, along with increased cell proliferation upon reoxygenation.

### 4.4. Phospholipid

Additional cell membrane components include phospholipid metabolism phospholipids (PLs), which are diversified in both structure and function. They are involved in homeostasis, cell adhesion and migration, signal transduction, vesicle transport, apoptosis and posttranslational modifications. Subclasses of PLs include phosphatidylcholine (PC), phosphatidylethanolamine (PE), phosphatidylserine (PS), phosphatidylglycerol (PG), phosphatidylinositol (PI), and phosphatidic acid (PA), depending on the type of polar head group.

Lysophosphatidylcholine acyltransferase (LPCAT) is gaining recognition as another class of enzyme that appears to be affected in many cancers. LPCAT1 upregulation is connected to clear renal cell carcinoma, oral squamous cell carcinoma, hepatoma, esophageal cancers, gastric cancers, breast cancers, colorectal cancers and prostate cancers. Prognosis and survival for clear cell renal cell carcinoma, breast cancer and prostate cancer are linked with LPCAT1 expression; it is useful as a diagnostic biomarker for prostate and esophageal cancers. Elevated levels of cell proliferation, migration and metastasis in clear cell renal cell carcinoma and HCC cell lines all result from overexpression of LPCAT1. Increased levels of saturated phospholipids are present in clear cell renal cell carcinoma, HCC and gastric cancers, which is aligned with LPCAT1 enzyme activity. Currently the relationship between the behavior of cancer cells and these types of changes in PC levels is now well understood and will require further research.

It is possible that tumor growth may be promoted when LPCAT1 undergoes lyso-PAF acetyltransferase activity to produce PAF, which is important to cell growth as a lipid mediator. Overexpression of LPCAT2 is seen in cervical and breast cancers and is thought to be a susceptibility gene for aggressive prostate cancer in animal models and genome-wide association studies in human patients. This indicates that by targeting LPCAT2-mediated intracellular LD formation, it may be possible to restore sensitivity to chemotherapy in colorectal cancer. When LPCAT3 is reduced in the mouse gut, it produces a reduction in the composition of polyunsaturated phospholipids and promotes tumor development and growth in ApcMin mice. LPCAT4 is associated with intestinal stem cell proliferation and tumorigenesis and is also associated with high levels of PC (16:0/16:1) in colorectal cancer.

### 4.5. Glycosphingolipids (GSLs)

GSLs are sphingophospholipids which contain glycosyl groups. Gangliosides are sialylated GSLs on the cell membrane of T cells which interact with pathogen antigens. Shigella, the enteroinvasive bacterium responsible for acute rectocolitis, binds to the CD4^+^ T cell via gangliosides; T cell activation is elevated in the infection mouse model as a result. HIV-1 cell infection is reduced when the ganglioside GM-1 restricts entry. Thus, bacterial and viral infection may be impaired through regulation of GSL metabolism.

## 5. Lipid Metabolism in Cancer Cells

There are a number of tumor-infiltrating immune cells, or TIIs, which are another component of tumors. Among them are T and B lymphocytes, tumor-associated macrophages (TAMs), dendritic cells (DCs), myeloid-derived suppressor cells (MDSCs), neutrophils and natural killer (NK) cells. Their functions vary from anti-tumor to tumor promoting depending on tumor stage and type. Changes in FA metabolism can alter the polarity of TAMs to a pro-tumoral phenotype. When tumor cells secrete macrophage colony-stimulating factor (M-CSF), FASN upregulation occurs. The result is activation of PPARβ/δ and expression of the anti-inflammatory cytokine IL-10.

## 6. Lipid Drug Candidates for Cancer

Table 6 shows agents as modulators of lipid metabolism in cancer [98,99,100,101,102,103,104,105,106,107,108,109,110,111,112,113,114,115,116,117,118,119,120,121,122]. Given that FAs are key in proliferation and progression of cancer cells, research into therapeutic strategies seeks drug candidates which act on lipogenic enzymes, transcription factors regulating the intracellular lipid homeostasis, or both. There are five target areas under consideration: (a) lipogenic enzymes (FASN, ACLY, ACC); (b) the exogenous lipid uptake (LXR, CD36, FABP4/5); (c) inflammatory signaling pathways (PTGS2); (d) regulation of intracellular lipid homeostasis (PPARγ, CPT1a, lipin2, HSL, MAGAT, DAGAT); and/or (e) saturated vs. unsaturated FAs. While in vitro preclinical and clinical cancer testing models have indicated the efficacy of these strategies, it must be noted that the varied regulatory mechanisms of lipid metabolism produce challenging side effects. A summary of the major drugs being evaluated is included herein.

## 7. Challenges for Lipidomics

- The unique nature, complexity and specificity of lipodomics research make it exciting and challenging. For example, mapping of lipidomes is not possible with present equipment and techniques, which are unable to provide the detailed information necessary to quantify individual lipid molecules present in an organism. The diversity of lipid classes and lipid molecular species complicates efforts for the structural identification of lipids by mass spectrometry and renders it impossible to accommodate all lipid classes with a common method for extraction, chromatography and detection. Several methods need to be applied.
- LC-MS is gaining favor over “shotgun” or direct infusion methods in lipodomics research, given its enhanced specificity and sensitivity. The greater specificity of MS/MS is invaluable for elucidating additional levels of detail which is crucial for identifying individual acyl chains.
- One of the challenges related to MS-based lipidomics is the complexity of the sample, with a small mass range between 300 and 900 Da. The classical quadrupoles have a limited mass resolution, and, usually, multiple lipid species with similar masses (referred to as isobaric species) are co-detected, hampering the exact assignment of a mass to a specific individual lipid species. The use of mass spectrometers with higher resolution of mass offers a partial solution to this issue. One example is the time-of-flight (TOF) analyzer, which uses the *m*/*z*-dependent acceleration of ions in a flight tube; another is the Orbitrap, where ions oscillate around an inner rod. Use of a Fourier transformation with the measured ion oscillations gives the Orbitrap excellent resolution of much smaller mass differences.
- The differences in ionization efficiency between species present a clear challenge in lipodomics research, though it is enhanced in some situations by the addition of salts such as ammonium acetate. Prior to extraction, one or multiple internal standards at known concentrations for each lipid class of interest are spiked.
- Reference standards and relevant internal standards remain limited.
- With the acquisition of the massive output of data generated, and subsequently requiring processing, the voracious demand for computer power and bioinformation clearly represents another major difficulty in MS-based lipodomics research. Spectral alignment and statistical evaluation of fluctuating signal intensities demands significant effort during the collection of chromatographic and MS data. The causes for these discrepancies include biological variations, sample handling issues and analytical accuracy; reliable resolution of lipid levels within a complex mixture may require additional replications.
- Software packages offer help for identifying correlations between lipid metabolites and physiological phenotypes, especially for developing lipid-based biomarkers. Given the extreme complexity of some lipid pathways, such as the mammalian glycosphingolipid pathway, information technology in lipodomics should help in constructing metabolic maps from data on lipid structures and lipid-related proteins and genes.
- There is a need to create searchable and interactive databases of lipids and lipid-related genes/proteins. Such databases can be integrated with MS and other experimental data, as well as with metabolic networks. These combined resources will lead to improved therapeutic strategies to prevent or reverse these pathological states involving dysfunction of lipid-related processes.

## 8. Common Software and Databases Used in Lipidomics

A list of common software databases and their websites used in lipidomics is shown below: All URLs were accessed on 14 March 2024

- METLIN, data analysis based on MS/MS spectra, ISF 0eV, https://enigma.lbl.gov/metlin-new-metabolite-identification/
- MZmine 3.3.0, LC-MS data analysis workflow, http://mzmine.sourceforge.net/
- Lipid View software 1.2 EULA, analysis based on electrospray MS data, https://sciex.com/products/software/lipidview-software
- LipidXplorer 1.2.8.1, data analysis based on MS and MS/MS spectra, https://lifs-tools.org/lipidxplorer.html
- LipidSearch 5.1 Software, automatically identify and relatively quantify LC-MS data, http://www.thermoscientific.com/content/tfs/en/product/lipidsearch-software.html
- LIPID MAPS, website, lipid structure, annotation, classification and pathways, analytical methods, http://www.lipidmaps.org
- Lipid Bank, data base, lipid structure, name, spectra and literature information, http://lipidbank.jp
- Lipid Library, lipid chemical, biological and analytical, http://lipidlibrary.co.uk
- LipidBlast, in silico tandem MS database for lipid identification, https://fiehnlab.ucdavis.edu/projects/lipidblast/
- CyberLipids, lipid structure and analytical methods, http://www.cyberlipid.org
- KEGG, synthesis and degradation of fatty acid, metabolic pathways of lipid, http://www.genome.jp/kegg/
- Metlin, MS/MS database, https://metlin.scripps.edu/landing_page.php?pgcontent=mainPage
- Cholesterol ester: http://ctdbase.org/detail.go?type=chem&acc=D002788&view=disease
- Glycosyltransferases, https://www.genenames.org/data/genegroup/#!/group/424
- Lipid Identification and Characterization using SimLipid. This is a high-throughput lipid identification and quantification software. It can process raw data from hundreds of LC-MS and MS/MS experimental runs for LC-peak detection, peak-picking, molecular feature finding and retention time alignment.

## 9. Conclusions

Herein, we present lipid classification and functions, and lipid extraction and analysis techniques used in lipodomics analysis, including tandem MS, MS/MS or MS^n^ [123,124,125,126]. We shed light on ways in which mass spectrometric technology has become much more efficient in characterizing and identifying molecular lipid species in total lipid extracts. Mass spectrometry benefits from its capability to use the mass-to-charge ratios (*m*/*z*) of charged ionized analytes for separation and characterization. Fragmentation of lipid ions by means of collision-induced dissociation (CID) generates structural information. These methods, which offer supreme selectivity, sensitivity and the ability to record these fragmentation reactions, provide structural information for components in complex mixtures. Using a targeted approach, as opposed to a global one, lipid analysis provides another method in lipidomics.

It has been reported that remodeling, reprogramming and metabolism to signaling are some of the pathways for cancer cells to undergo profound alterations in lipid homeostasis. T cell differentiation, functions and survival are increasingly demonstrated to be linked to processes of lipid metabolism. Table 7 shows lipid metabolism in functional subsets of T cells. In this review, we have shown that lipidomics provides a promising and potentially powerful tool for the development of new drugs which are targeted to treat cancers. Also, analyzing the relative lipid content in tumor and normal tissue microenvironments is proving helpful in narrowing our search for more precisely targeted lipid metabolism regulation strategies. In addition to its role as an essential component of cancer cell growth and survival, lipid metabolism is involved in the crosstalk with immune cells in the TME. As we improve our knowledge of mechanisms of lipid metabolism in cancer, we seek to gain a better understanding of related enzymes and genetic factors contributing to their metabolism. This knowledge offers the potential for the development of newer, more powerful and precise treatments to fight cancer and restore tumor immunology.

## Figures and Tables

**Table 1:** Examples of lipids categories.

Categories	Classes	Example
Fatty acyls, FA	Fatty acids: straight-chain fatty acids, Eicosanoids, fatty alcohols, fatty esters, fatty amides	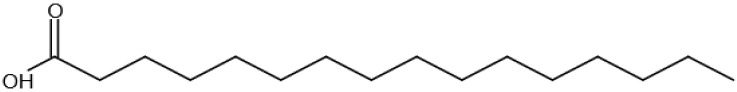 Hexadecanoic acid
Prenol lipids, PR	Isoprenoids, Quinones and hydroquinones, Polyprenols	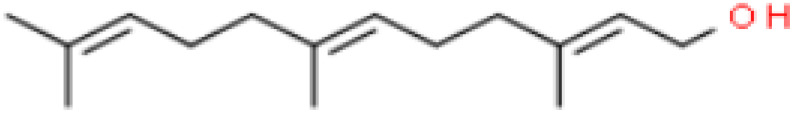 2E,6E-Farnesol
Glycerolipids, GL	Monoradylglycerols: monoacyl glycerols, Diradylglycerols: diacyl glycerols, Triradylglycerols: triacyl glycerols	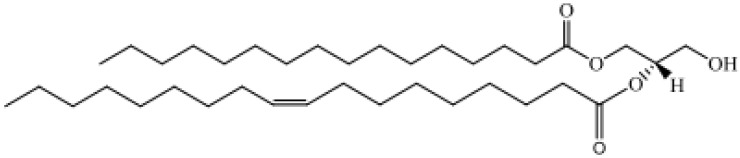 1-Hexadecanoyl-2-(9Z-octadecenoyl)-sn-glycero
Glycerophospholipids, GP	Glycerophosphocholines, glycerophosphoenolamines, glycerophosphoserines, glycerophospholycerols, glycerophosphoglycerophosphates, glycerophosphoinositols, glycerophosphoglycerophosphoglycerols	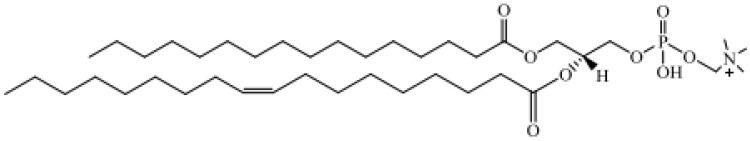 1-Hexadecanoyl-2-(9Z-octadecenoyl)-sn-glycero-3-phosphocholine
Sphingolipids, SP	Sphingoid bases, ceramides, phosphosphingolipids, neutral glycosphingolipids, acidic glycosphingolipid	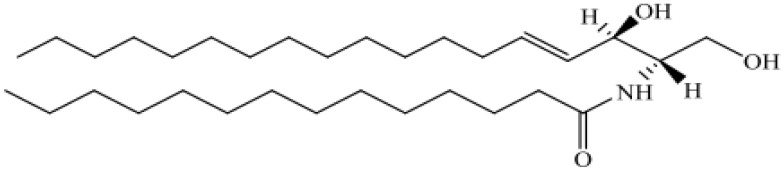 N-(Tetradecanoyl)-sphing-4-enine
Saccharolipids, SL	Acylaminosugars, Acylaminosugar glycans, Acyltrehaloses, Acyltrehalose glycans	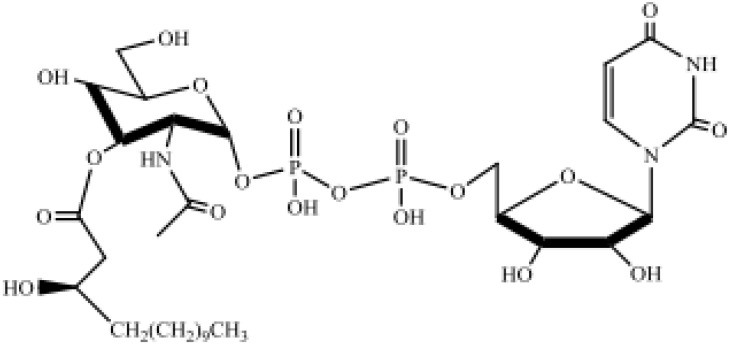 UDP-3-O-(3R-Hydroxyl-tetradecanoyl)-αD -N-acetylglucosamine
Sterol lipids, ST	Sterols, cholesterol and derivatives, steroids, bile acids and derivatives	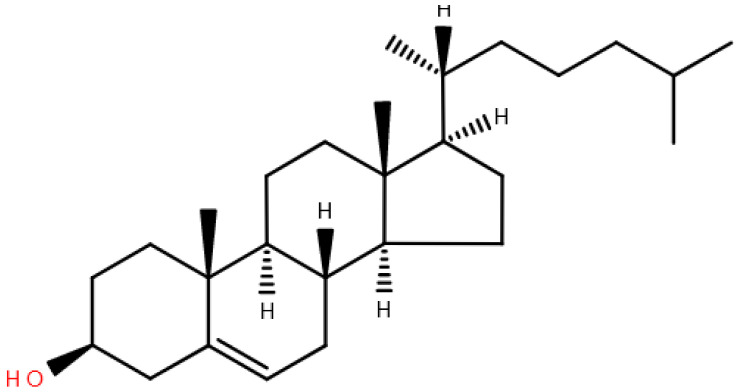 Cholest-5-en-3β-ol
Polyketides, PK	Macrolide polyketides, Aromatic polyketides, Nonribosomal peptide/polyketides hybrids	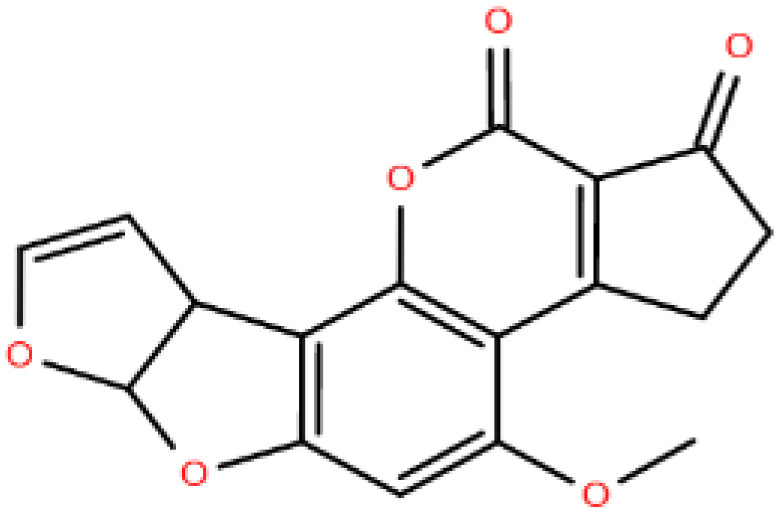 Aflatoxin B1

Adapted with modifications from [11].

**Table 2:** Major functions of lipid classes.

Membrane components	Glycerophospholipids (e.g., PC, PE, PI, PS, PG, PA, etc.), sphingolipids (e.g., sphingomyelin, cardiolipin, cerebroside, sulfatide, gangliosides, etc.), glycolipids, sterol lipids (e.g., cholesterol, etc.)
Energy storage and metabolism	Glycerolipids (e.g., NEFA, TAG, diacylglycerol, monoacylglycerol, acyl CoA, acylcarnitine, etc.)
Signaling	Glycerolipids (e.g., diacylglycerol, monoacylglycerol, acyl CoA, acylcarnitine, NEFA, oxidized fatty acid), sphingolipids (e.g., Cer, sphingosine, sphingosine-1-phosphate), psychosine, N-acylethanolamine, Lyso-lipids, etc.
Other functions	Plasmalogen (antioxidant) Acylcarnitine (lipid transport) CL (mitochondrial respiration), PS (cofactor, substrate of PE synthesis)

Choline glycerophospholipid (PC), ethanolamine glycerophospholipid (PE), phosphatidylinositol (PI), phosphatidylglycerol (PG), phosphatidic acid (PA), phosphatidylserine (PS), sphingomyelin (SM), cardiolipin (CL), Galactosylceramide (GalCer), Glucosylceramide (GluCer), non-esterified fatty acid (NEFA), triacylglycerol (TAG), diacylglycerol (DAG), monoacylglycerols (MAG), Eicosanoids and other oxidized fatty acids, sphingoid-1-phosphate (S1P).

**Table 4:** Lipidomic studies on biofluids reporting diagnostic lipid signatures.

Cancer	Lipids Modified	Technique
Ovarian	PPE(16:0, 18:1), LPC15:0 LPA18:2, PPE(18:0, 22:6)	LC/ESI/MS/MS
Liver	LPC16:0 LPC18:0 PS16:0, PC18:0	UPLC-MS
Liver	18:2n-6, 18:1n-9, 20:4n, 16:0	GC-MS
Lung	SM(16:0, 16:1), LPC18:1 LPC20:4 LPC20:3 LPC22:6	FTICR-MS
Lung	LPE18:1 CE18:2 ePE40:4, SM22:0	ESI-MS
Prostate	ePC38:5 PC40:3, PC42:4	Q-TOF-MS
Breast	Palmitic acid, 16:0, Stearic acid 18:0, Linoleic acid, 18:2, Alpha-Linolenic acid, 18:3, Eicosapentaenoic acid 20:5, Docosapentaenoic acid 22:5	GC-MS
Colorectal	Capric acid, 10:0	GC-MS

Adapted with modifications from [46].

**Table 5:** Potential lipid biomarkers in the oncology field.

Tumor	Lipid Class	Lipid Type	Sample	Up	Down	Ref.
Lung cancer (adenocarcinomas)	GPLs	PI arachidonate containing phospholipids	Tissue	X		[47]
Lung cancer (adenocarcinomas)	GPLs	PC 32:0, PC 32:1, PGs	Tissue		X	[47]
Lung cancer (adenocarcinomas)	FAs	free arachidonic	Tissue	X		[47]
Lung cancer (NSCLC)	GPLs	PC 34:1, PC 36:2, PC 36:3	Tissue	X		[48]
Lung cancer (NSCLC)	GPLs, SLs	PC 32:0, ST-OH 42:1	Tissue	X		[48]
Lung cancer (NSCLC)	FAs	EPA	Tissue	X	X	[49]
Lung cancer (Different cancer type)	GPLs	SM 16:0/1, LPC 18:1, LPC 20:4, LPC 20:3, LPC 22:6	Serum	X		[50]
Lung cancer (NSCLC)	GPLs	PI 38:3, PI 40:3, PI 38:2	Tissue	X		[51]
Lung cancer (NSCLC)	SLs	SM 40:1, SM 42:1, SM 36:1	Tissue		X	[51]
Breast cancer NS	GPLs, SLs	PC, PE, PI, SMs	Tissue	X		[52]
Breast cancer (luminal, HER2+ and triple-negative)	GPLs	PC 34:1	Tissue	X		[53]
Breast cancer (MDA-MB 231 model)	Palmitoyl carnitines, stearoyl carnitine GPLs, SLs	PC16:0/16:0,PC16:0/18:1, PC18:1/18:1,PC18:0/18:1 PC16:0/22:1 SMd18:1/16:0	Tissue	X		[54]
Breast cancer NS	GPLs	PI18:0/20:4	Urine		X	[55]
Breast cancer NS	GPLs	PS (18:1/18:1 18:2/18:0)	Urine	X		[55]
Breast cancer NS	GPLs	PI 18:0/18:1, PI 18:0/20:3	Tissue	X		[56]
Breast cancer (mammary epithelial and breast cancer)	GPLs	PCs, PI 22:5/18:0, PI 18:0/18:1	Cell line	X	X	[57]
Breast cancer NS	GPLs	PS 18:0/20:4, PI 18:0/20:4, PC 18:0/20:4	Cell line	X		[58]
Breast cancer NS	GPLs	PIs, Pes, PCs, LPCs	Tissue	X	X	[59]
Breast cancer NS	GPLs	TGs containing C18:1 fatty acyl chain	Serum		X	[60]
Breast cancer NS	FAs	Linoleic acid (C18:2)	Serum		X	[61]
Prostate cancer NS	GPLs	PS 18:0/18:1, PS 16:0/22:6	Urine	X		[62]
Prostate cancer NS	GPLs	PS 18:1/18:0, PS 18:0/20:5	Urine		X	[62]
Prostate cancer NS	GPLs	PI 18:0/18:1, PI 18:0/20:3, PI 18:0/20:2	Tissue	X		[63]
Localized prostate cancer	GPLs	LPC 16:0/OH, SM d18:1/16:0	Tissue		X	[64]
Localized prostate cancer	GPLs	LPC 16:0/OH	Tissue		X	[64]
Localized prostate cancer	GPLs	PC 40:3, PC 42:4	Serum	X		[65]
Prostate cancer NS	GPLs	PC 39:6	Serum	X		[66]
Prostate cancer NS	FAs	FA 22:3	Serum	X		[66]
Colorectal cancer NS	GPLs	LPC 18:1, LPC 18:2	Plasma		X	[67]
Colorectal cancer (pT3 stage, various grades (G2, G3))	GPLs	PC/PE ratio	Cell lines	X		[68]
Colorectal cancer NS	GPLs	PC 16:0/16:1	Tissue	X		[69]
Colorectal cancer NS	GPLs	PC 16:0/18:1, LPC 16:0, LPC 18:1	Tissue	X		[70]
Colorectal cancer liver metastasis	GPLs	PE 38:6, PE 40:4	Tissue	X		[71]
Colorectal cancer NS	FAs	n-3 PUFAs	Red Blood cell		X	[72]
Colorectal cancer NS	FAs	n-6-PUFA/n-3-PUFA	Red Blood cell	X		[72]
Ovarian cancer NS	GPLs	LPC	Plasma	X		[73]
Ovarian cancer NS	GPLs	PC, TG	Plasma		X	[73]
Epithelial ovarian cancer	GPLs	TGs 50:2 50:152:2 54:4 54:3	Cell lines	X		[74]
Ovarian cancer NS	GPLs	PC 32:3, PC 34:1, PC 36:2	Tissue	X		[75]
Ovarian cancer NS	GPLs	LPA 16:0, LPA 20:4	Plasma	X		[76]
Ovarian cancer and other gynecological cancers	GPLs	LPA	Serum/plasma	X		[77]
Ovarian cancer and other gynecological cancers	GPLs	LPA 16:0, LPA 18:2, LPA18:1, LPA18:0, LPI 16:0, LPI 18:0, LPI 20:4	Plasma	X		[78]
Benign and malignant ovarian cancer	GPLs	LPA	Plasma	X		[79]
Benign and malignant ovarian cancer	GPLs	LPA	Plasma	X		[80]
Benign and malignant ovarian cancer	GPLs	Plasmalogen phospatidylethanol, PC, plasmalogen PC, SM and LPC		X		[81]
Metastatic pancreatic cancer	SLs	Ceramides species (C16:0 and C24:1)	Tissue/Plasma	X		[82]
Metastatic pancreatic cancer	SLs	C18:0 C20:0 C22:0 C24:0 C24:1	Tissue/Plasma		X	[82]
Metastatic pancreatic cancer	SLs	C16:0 C20:0 C22:0 C24:0 C24:1	Tissue/Plasma	X		[82]
Pancreatic cancer PANC-1 cells	GPLs	LPA	Cell lines	X		[83]
Pancreatic cancer NS	FAs	MUFA	Plasma	X		[84]
Gastric cancer NS	GPLs	PC16:0/18:1	Tissue	X		[85]
Gastric cancer NS	GPLs	LPC 16:0	Tissue		X	[85]
Bladder Cancer NS	GPLs	PS 18:0/18:1	Tissue	X		[86]
Bladder Cancer	GPLs	PI 18:0/20:4	Tissue	X		[86]
Bladder Cancer (Model of human invasive bladder cancer)	GPLs	PS 18:0/18:1	Tissue	X		[87]
Bladder Cancer (Model of human invasive bladder cancer)	GPLs	PG 18:1/18:1	Tissue	X		[87]
Bladder Cancer (Model of human invasive bladder cancer)	GPLs	PI 16:0/18:1	Tissue	X		[87]
Bladder Cancer (Model of human invasive bladder cancer)	GPLs	PI 18:0/18:1	Tissue	X		[87]
Bladder Cancer (Model of human invasive bladder cancer)	GPLs	PS 18:1/18:1	Tissue	X		[87]
Esophageal cancer (ESCC)	GPLs	Octanoylcarnitine, LPC 16:1, Decanoylcarnitine	Plasma	X		[88]
Esophageal cancer (OSCC)	GPLs	PC 16:0/16:1	Tissue	X		[89]
Esophageal cancer (OSCC)	GPLs	PC 18:1/20:4	Tissue		X	[89]
Esophageal cancer (ESCC)	GPLs	PS, PA, PC, PI, PE	Plasma	X		[90]
Kidney cancer NS	GLs SLs	PE (P-16:0e/0:0), ganglioside GM3, (d18:1/22:1), sphinganine C17, SMd18:0/16:1(9Z)	Serum	X		[91]
Kidney cancer NS	GPLs STLs Gls	PC, Plasmalogens, cholesterol esters, TGs	Tissue	X		[92]
Kidney cancer NS	GPLs, FAs	PE, Unsaturated FAs	Tissue		X	[92]
Kidney cancer NS	GPLs	PL, PE 36:1, PC 38:4, PC 36:2, PC 32:0	Tissue	X		[93]
Kidney cancer NS	GPLs	PE 34:2, PE 36:4, PE 38:4, PC 34:1 PC34:2 PC 36:4 PI36:4	Tissue		X	[93]
Kidney cancer NS	GPLs	PI 18:0/20:4, PI22:4/18:0 PS18:0/18:1 PG18:1/18:1	Tissue	X		[94]
Kidney cancer (Human papillary renal carcinoma)	FAs	FA12:0	Tissue		X	[94]
Thyroid cancer (Thyroid papillary cancer)	GPLs	PC 16:0/18:1 PC 16:0/18:2	Tissue	X		[95]
Thyroid cancer (Thyroid papillary cancer)	SLs	SMd18:0/16:1	Tissue	X		[95]
Malignant and benignant thyroid cancer	GPLs	PC 34:1, PC 36:1, PC 32:0	Tissue/Serum	X		[96]
Malignant and benignant thyroid cancer	GPLs	PA 36:62, PA 36:3, PA 38:4, PA 38:5, PA 40:5	Tissue/Serum	X		[96]

Adapted with modifications from reference [97].

**Table 6:** Overview of potential agents as modulators of lipid metabolism in cancer.

Target	Agent Name	Type of Cancer/Phase of Development	Comments and Benefits	Reference/Clinical Trial
Fatty acid synthase (FASN) inhibitor	Orlistat	Prostate, breast, ovarian, colon cancer and other solid tumors/Approved	Antiobesity lipase and FASN inhibitor; not yet assessed clinically for cancer. An anti-obesity drug approved by the FDA and an irreversible inhibitor of FASN	[98]
	TVB-3166/TVB-3664	Oral squamous cell carcinoma, colorectal, breast cancer/Preclinical	A reversible and selective FASN inhibitor	[99,100]
	Conjugated Linoleic Acid	Breast Cancer/Phase I	Reduce *FASN* gene expression and spot 14	NCT00908791
	Omeprazole	Triple-negative breast cancer/Phase II	A proton pump inhibitors that can inhibit FASN	NCT02595372
	IPI-9119	Castration-resistant prostate cancer/Preclinical	Selectively inhibit FASN and suppress expression of both full-length of androgen receptor (AR) and AR variant V7	[101]
	TVB-2640	Solid Malignant Tumor/Phase II	Trials in breast cancer +/− trastuzumab/docetaxel. A potent and reversible FASN inhibitor	NCT03032484 NCT03179904
Acetyl-CoA Carboxylase (ACC)	TOFA	Lung cancer and colon carcinoma/Preclinical	Induce apoptosis as an allosteric inhibitor of ACC-alpha	[102]
	Soraphen A	Prostate cancer/Preclinical	Inhibit fatty acid synthesis and stimulate fatty acid oxidation	[103]
	ND-646	NSCLC/Preclinical	Inhibit fatty acid synthesis and tumor growth as an allosteric inhibitor of the ACC	[104]
3-hydroxy-3-methylglutaryl coenzyme A reductase (HMGCR)	Statins, e.g., lovastatin, atorvastatin, rosuvastatin and simvastatin	Various leukemia and solid tumors/Phase II	Inhibitors of HMGCR. Association studies between statin use and outcomes in many cancers, some randomized trials	NCT03358017 NCT03324425 NCT02569645 NCT03275376
CD36	ABT-510	Prostate cancer/Phase II	Trial in metastatic melanoma. Reduced fatty acid uptake and the abundance of oncogenic signaling lipids	[105]
SREBPs	Fatostatin	Prostate cancer/Preclinical	A non-sterol diarylthiazole derivative which has antimitotic properties and perturbs nuclear translocation of SREBP and androgen receptor signaling	[106]
	Betulin	BRAF^V600E^-mutant melanoma/Prelinical	Increase membrane lipid poly-unsaturation and lipid peroxidation; sensitize therapy-resistant melanoma cells to MAPK-targeting therapy	[107]
	PF-429242	HCC/Preclinical	Reversible site 1 protease inhibitors, which inhibit endogenous SREBP processing	[108]
AMPK	MT 63–78	Prostate cancer/Preclinical	*MT 63–78* is a specific and potent direct AMPK activator and also induces cell mitotic arrest and apoptosis	[109]
	5-aminoimidazole 4- carboxamide riboside (AICAR)	Prostate cancer/Preclinical	Preclinical studies in cancer and an AMPK activator which inhibits cell growth	[109]
LXR	LXR-623	Phase I for atherosclerosis	Preclinical studies in cancer	[110]
	GW3965; LXR623	Melanoma and glioblastoma/Preclinical	LXR agonists which suppress mitochondrial respiration and decrease cholesterol levels by enhancing the excretion and decreasing the resorption of cholesterol	[111]
ATGL	atglistatin		Specific for murine ATGL	
MAGL	JZL184	Cancer-associated bone disease/Preclinical	Potent and reversible MAGL inhibitors	[112]
SCD	A939572	Glioblastoma and renal cell carcinoma/Preclinical	Inhibit tumor growth both in vitro and in vivo; overcoming chemotherapy agent resistance	[113]
	CVT-11127 or CVT- 12012	Lung cancer/Preclinical	A small molecule SCD inhibitor which modulates cancer cell metabolism, proliferation and survival	[114]
	MF-438	Lung cancer/Preclinical	Induce lung cancer stem cell apoptosis	[115]
CPT1	Etomoxir	Glioma/Preclinical	A CPT1 inhibitor which inhibits proliferative activity	[116]
	Perhexiline	Breast and gastrointestinal cancer/Preclinical	A CPT1 inhibitor which blocks FFA utilization, OxPhos and proliferation	[117]
PLD	ST1326	Preclinical		
	FIPI	Preclinical	Targets immune infiltration into tumors	
	VU0155072–2	Preclinical	Targets immune infiltration into tumors	
FABP	EI-05	Preclinical		
	SBFI-102, 103	Preclinical		
Choline kinase	HC-3, JCR and MN derivatives	Preclinical		
	Triterpene quinone methides	Preclinical	Derived from natural products	
	ICL-CCIC-0019	Preclinical		
	EB-3D, EB-3P	Preclinical		
Farnesyltransferase	tipifarnib	Phase I/II	Phase II studies in metastatic BC and squamous cell carcinoma; Phase I trial in glioblastoma	
	lonafarnib	Phase I/Ib	Largely combination studies in glioblastoma, BC	
	BMS-214662	Phase I	Single agent or in combination with paclitaxel in advanced solid tumors	
Palmitoylation	2-bromopalmitate	Preclinical		
Acid sphingomyelinase	Fluphenazine	Preclinical		
Mammalian target of rapamycin (mTOR)	Rapamycin	Breast cancer/Preclinical	Inhibited S6 phosphorylation and cell proliferation, and resulted in lower levels of apoptosis induction	[118]
	Everolimus	Castrated Resistant Prostate Cancer. Locally Advanced Cervical Cancer/Phase III	Directly inhibit mTORC1 and indirectly inhibit mTORC2	NCT03580239
	PF-05212384	Advanced Cancer; Advanced squamous cell lung, pancreatic, head and neck, and other solid tumors/Phase I	Intravenous PI3K/mTOR inhibitor	NCT01347866 NCT03065062
	PF-04691502	Breast Neoplasms/Phase II	Inhibit PI3K and mTOR kinase	NCT01658176
	Vistusertib/AZD2014	Endometrial, triple negative breast cancer, ovarian, primary peritoneal, or fallopian tube cancer/Phase II	mTORC1/2 Inhibitor	NCT02208375
Protein kinase B (PKB), Akt	MK-2206	Advanced or metastatic solid tumors or breast cancer; prostate cancer/Phase I, II	Inhibit Akt phosphorylation, cell proliferation and apoptosis in a dose-dependent manner	NCT01245205 NCT01277757 NCT01251861
	Capivasertib/AZD5363	Breast cancer, prostate cancer and advanced solid tumors/Phase I, II	A novel pan-AKT kinase catalytic inhibitor	NCT03310541 NCT02525068
	GSK2141795	Endometrial cancer/Phase I	AKT inhibitor	NCT01935973
ApoA-I	Apabetalone (RVX-208)	Colorectal cancer/Preclinical	A BET inhibitor which is a stimulator of ApoA-I and regulates the reverse cholesterol transport	[119]
	ApoA-1 mimetic peptides	Preclinical	Mimetic peptides which are synthesized on the basis of aamphipathic helical repeating structure of ApoA-I	[120]
ACAT	K604	Glioblastoma/Preclinical	A selective ACAT1 inhibitor, which suppresses proliferation of glioblastoma cells	[121]
	ATR-101	Advanced adrenocortical carcinoma/Phase I	An oral selective ACAT inhibitor	NCT01898715 [122]

**Table 7:** Lipid metabolism in functional subsets of T cells.

T-Cell Subset		Metabolic Events	Molecular Players
Naïve T cell		Majorly rely on OXPHOS and FAO	Enzymes of OXPHOS and FAO
Ag- stimulated T cells		Aerobic glycolysis, FA synthesis, uptake, and accumulation	mTOR, PI3K, c-myc, HIF, GLUTs, FASN, SREBP, and PPARg
CD8+ cytotoxic T cells		Mainly rely on FA and lipid synthesis	FASN, ACC1, HMGCR, and SREBP
CD4+ helper T cells		FA and lipid synthesis and FA uptake	ASN, SREBPs, CD36, FABPs, GPR43, GLP84, and LXR
	Th1	Relatively high FA uptake dominates over FA synthesis	CD36, FABPs, FASN, and LCFA favor Th1 activation
	Th2	Relatively low FA uptake, however, dominates over FA synthesis	CD36, FABPs, FASN, and PUFA favor Th2 activation
	Th3	Profound FA synthesis and aerobic glycolysis	FASN, ACC1, PDHK, LXR, and 2-HG favor Th17 activation
Regulatory T cells (Treg)		Exogenous FA uptake dominates over FA synthesis, OXPHOS, and FAO	Foxp3, FABP5, CPT1, downregulation of ACC1, SREBPs, and SCAP
Memory T cells (Tm)		OXPHOS and FAO, FA uptake, and downregulation of FA synthesis	Enzymes of OXPHOS and FAO, FABP4/5, AMPK, and downregulation of ACC1

OXPHOS, oxidative phosphorylation; FAO, fatty acid oxidation; FA, fatty acid. Adopted from reference [122].

## Data Availability

Not Applicable.
